# Divulgación accesible de la ciencia

**DOI:** 10.31053/1853.0605.v80.n3.42305

**Published:** 2023-09-29

**Authors:** L. Johana Escobar Zuluga

**Affiliations:** 1 Universidad Nacional de Córdoba. Facultad de Ciencias Médicas. Consejo Nacional de Investigaciones Científicas y Técnicas. Instituto de Investigaciones en Ciencias de la Salud Argentina

**Keywords:** publicaciones de divulgación científica, materiales educativos y de divulgación, e-accesibilidad, science outreach publications, educational and outreach materials, e-accessibility, publicações de divulgação científica, materiais educativos e de divulgação, acessibilidade eletrónica

## Abstract

Cuando reconocemos la importancia de la divulgación del conocimiento científico, reconocemos no solo el potencial que este puede llegar a tener en manos de la comunidad científica sino también de toda la sociedad. Desde esa premisa primero reflexionamos, esperando que tal reflexión nos lleve a comprometernos a que las acciones de divulgación sean accesibles y no discriminatorias. “La igualdad de acceso a la ciencia no sólo es una exigencia social y ética para el desarrollo humano, sino que además constituye una necesidad para explotar plenamente el potencial de las comunidades científicas de todo el mundo y orientar el progreso científico de manera que se satisfagan las necesidades de la humanidad".

## Abstract (en)

When we recognize the importance of disseminating scientific knowledge, we recognize not only the potential it can have in the hands of the scientific community, but also in the hands of society as a whole. From this premise, we first reflect, hoping that such reflection will lead us to commit ourselves to make dissemination actions accessible and non-discriminatory. "Equal access to science is not only a social and ethical requirement for human development, but also a necessity to fully exploit the potential of scientific communities around the world and to guide scientific progress in a way that meets the needs of humanity".

## Resumo

Quando reconhecemos a importância da divulgação do conhecimento científico, reconhecemos não só o potencial que este pode ter nas mãos da comunidade científica, mas também nas mãos da sociedade em geral. Partindo desta premissa, começamos por refletir, esperando que essa reflexão nos leve a empenharmo-nos em tornar as acções de divulgação acessíveis e não discriminatórias. "A igualdade de acesso à ciência não é apenas uma exigência social e ética para o desenvolvimento humano, mas também uma necessidade para explorar plenamente o potencial das comunidades científicas de todo o mundo e para orientar o progresso científico de forma a satisfazer as necessidades da humanidade".

Para aquellos que están en el campo de la investigación científica, le son conocidas las amplias y variadas contribuciones que esta puede llegar a tener para la sociedad. Si recordamos, una de las reflexiones más significativas de la "Declaración sobre la ciencia y el uso del saber científico" (Declaración de Budapest, 1999), es precisamente que, "la investigación científica y sus aplicaciones pueden tener repercusiones considerables con vistas al desarrollo humano sostenible, comprendida la mitigación de la pobreza, y que el futuro de la humanidad dependerá más que nunca de la producción, la difusión y la utilización equitativa del saber"^(1)^. Se podría entender que, el proceso de investigación y generación del conocimiento no estará completo, si no se realiza la generalización de los resultados obtenidos. No estará completa si no llega a todos los colectivos. Es aquí donde reconocemos no solo la importancia de la divulgación, entendida como el proceso a través del cual un conjunto de conocimientos llega a muchas personas, sino también de que esta sea accesible y no discriminatoria. En esta misma conferencia, se establece que, "la igualdad de acceso a la ciencia no sólo es una exigencia social y ética para el desarrollo humano, sino que además constituye una necesidad para explotar plenamente el potencial de las comunidades científicas de todo el mundo y orientar el progreso científico de manera que se satisfagan las necesidades de la humanidad" ^1^ .

Sabemos también que son varios los desafíos en términos de divulgación científica, no solamente el tema de la accesibilidad. El primero de ellos, que excede muchas veces los alcances de nuestro actuar como investigadores/as, es la baja cultura científica que existe en nuestros medios. Cultura referida al grado de presencia de la ciencia y la tecnología en la sociedad. Acá se hace necesario, tal como afirma Diego de Arriba (2007), "Promover una cultura científico-tecnológica que convierta a la ciudadanía en mediadora eficaz entre la ciencia, la tecnología y la sociedad, capaz de comprender e interpretar aquéllas desde una perspectiva social" ^2^ , desarrollando así capacidades para transformar la sociedad en la que vive. Lo que nos y los convertiría en actores mucho más activos que pasivos. En segundo lugar, vale la pena hacer la reflexión sobre la poca difusión que se hace de los proyectos de investigación llevados a cabo o de sus resultados. Loaiza señala, en un estudio realizada entre los años 2008 y 2009, en 5 centros de investigación científica que, "solo la mitad del personal científico participa en actividades de comunicación pública de la ciencia, o lo hacen regularmente". Afirma, además que, los y las científicas tienen dificultades para conectar con el público. Las dificultades se deben, sobre todo, a la falta de apoyo institucional, las presiones laborales y la falta de una formación en comunicación ^3^ . En este punto vale la pena hacernos algunas preguntas. ¿Qué es comunicar ciencia?, ¿Por qué y para que hacerlo?, ¿Para quienes estamos comunicando?, y ¿Para quienes deberíamos hacerlo? Muchas veces en las instituciones de ciencia y tecnología y quienes las conforman, sigue predominando una lógica académica individualista, enfocada principalmente en ampliar la frontera del conocimiento. Simanauskas afirma que, hay una desconexión, claramente, entre este enfoque academicista, individualista y la necesidad de la resolución de los problemas sociopolíticos, ambientales y locales de nuestras sociedades latinoamericanas o de otros países del mundo. En otras palabras, lo que ocurre es que hay un desacople, entre el mundo académico, el mundo de la producción del conocimiento y la calle, por así decirlo, lo que está por fuera ^4^ .

Ahora hablando más específicamente acerca de que tan accesible son estos contenidos, sean artículos, resúmenes, contenido audiovisual, etc. Es necesario pensar, por ejemplo, en algunas barreras presentes para el colectivo de personas con discapacidad o con diversidad funcional. Cintia Ibalvaz, Lic.en Educación para la Salud, becaria de la fundación ICSA, quien presenta una discapacidad visual, hace algunas reflexiones al respecto. "La necesidad de promover una cultura científica presenta desafíos, pero estos se ven incrementados en el caso del colectivo de personas con discapacidad, por las dificultades y obstáculos que persisten tanto para su acceso a la educación, como a los medios de información científica. Las personas con discapacidad que están en el mundo de la academia o las ciencias o aquellas que desean estar, no solamente queremos el resumen del artículo que está en algún formato accesible, deseamos poder leer todo el artículo. Y esto a veces no es posible, ya que cuando logramos adaptar el PDF (formato en el que se encuentran los artículos) a un formato accesible para nosotros, necesitamos de alguien que nos detalle el contenido de imágenes, gráficos, tablas, que la mayoría de veces no están descritos en su totalidad" ^5^ . Para finalizar, es importante pensar, tal como afirman Toboso y Guzmán, que, "El logro de la accesibilidad requiere de una estrategia activa para realizarse, lo que nos lleva al concepto de "diseño para todos". El diseño para todos se define como "la actividad por la que se conciben o proyectan, desde el origen y siempre que ello sea posible, entornos, procesos, productos, servicios, objetos, instrumentos, dispositivos o herramientas, de tal forma que puedan ser utilizados por todas las personas independientemente de su edad y de sus capacidades funcionales (físicas, psíquicas y sensoriales)" ^6^ . El desafío esta entonces en como tener presente este concepto de "diseño para todos" en nuestras acciones de divulgación científica o comunicación de las ciencias. Vale la pena, conocer las herramientas disponibles para esto y acercarnos a algunas iniciativas exitosas que en algunas partes o de la Argentina o del mundo se vienen consolidando, esta es la invitación.

Algunas iniciativas exitosas:

Ciencias sin barreras: http://cienciasinbarreras.theiagd.org/actividades

Geodivulgar: https://www.ucm.es/geodivulgar/

Astronomía inclusiva: https://astronomia.udp.cl/es/the-advantages-of-making-science-accessible/
